# Comparative analysis of inflammatory biomarkers for the diagnosis of neonatal sepsis: IL-6, IL-8, SAA, CRP, and PCT

**DOI:** 10.1515/biol-2022-1005

**Published:** 2025-01-28

**Authors:** Ying Chen, Aixia Yan, Li Zhang, Xiaoming Hu, Liang Chen, Jun Cui, Zichuan Fan, Ying Li

**Affiliations:** Department of Neonatology, Children’s Hospital, Capital Institute of Pediatrics, 2 Yabao Road, Chaoyang District, Beijing, 100020, China; Chinese Academy of Medical Sciences & Peking Union Medical College, Beijing, 100730, China; Department of Pediatrics, Aerospace Central Hospital, Beijing, 100012, China

**Keywords:** IL-6, IL-8, SAA, neonatal sepsis, diagnosis, ROC curve

## Abstract

Neonatal sepsis (NS) is highly likely to cause death; however, early diagnosis of NS is still a great challenge. This study aimed to determine the diagnostic values of IL-6, IL-8, and serum amyloid A (SAA) in NS patients. C-Reactive protein (CRP), procalcitonin (PCT), interleukin (IL)-6, IL-8, and SAA were detected in 120 infants with NS (60 premature infants [NS-PIs] and 60 term infants [NS-TIs]). Sixty noninfected premature infants and 60 noninfected term infants composed the control group. Receiver operating characteristic (ROC) curves were used to determine the sensitivity and specificity of IL-6, IL-8, and SAA alone and in combination with CRP and PCT. The area under the curve (AUC) was calculated to evaluate the diagnostic value. The clinical characteristics of the subjects were recorded. The expression of CRP, PCT, IL-6, IL-8, and SAA was upregulated in patients with NS compared with control subjects. When the SAA cut-off value was 10.18 mg/L, the greatest AUC for the diagnosis of NS-PIs was for SAA (AUC = 0.833, 95% CI 0.762–0.905, *P* < 0.001). When the CRP cut-off value was 9.562 mg/L, the smallest AUC for the diagnosis of NS-PIs was for CRP (AUC = 0.776, 95% CI 0.684–0.867, *P* < 0.001). When the IL-8 cut-off value was 52.03 pg/mL, the greatest AUC for the diagnosis of NS-TIs was for IL-8 (0.821). When the IL-8 cut-off value was 52.03 pg/mL, the greatest AUC for the diagnosis of NS-TIs was for IL-8 (AUC = 0.821, 95% CI 0.745–0.898, *P* < 0.001). When the CRP cut-off value was 13.18 mg/L, the smallest AUC for the diagnosis of NS-TIs was for CRP (AUC = 0.762, 95% CI 0.667–0.857; *P* < 0.001). Additionally, according to the AUC value, the best combination was SAA and PCT for NS-PI diagnosis, and the best combination was PCT and IL-6 for NS-TI. In conclusion, compared with PCT and CRP, IL-6, IL-8, and SAA are better diagnostic biomarkers. Moreover, PCT combined with SAA is more suitable for diagnosing NS-PIs, and PCT combined with IL-6 is more suitable for diagnosing NS-TIs.

## 1 Introduction

Neonatal sepsis (NS) is a serious systemic bloodstream infection that originates from intrauterine infection or local infection, endangering the health and life of newborns [1]. The immature immune system of newborns results in an impaired response to infectious agents, especially in premature infants [2]. At present, the morbidity of NS is approximately 2%, with mortality ranging from 11 to 19% [3]. NS can be divided into two types: early-onset NS (within 72 h of birth) and late-onset NS (72 h after birth) [4]. The clinical management of NS is complicated by diverse causative pathogens and varying susceptibility across individuals [5,6]; thus, early diagnosis is very important. However, owing to the diverse clinical features of NS and a lack of typical symptoms and obvious early manifestations, the rapid and accurate diagnosis of NS remains a great challenge.

C-Reactive protein (CRP) is an acute inflammation-related protein, and its abundance increases up to 1,000-fold in response to infection or inflammation [7]. CRP is produced mainly by liver cells but can also be produced by smooth muscle cells, macrophages, and endothelial cells. Given that CRP levels return to baseline upon patient improvement, CRP is a useful marker to monitor infection status [8]. CRP has been used for the early detection of NS. Procalcitonin (PCT) is the precursor of the hormone calcitonin. PCT responds to infection faster than CRP and begins to increase at 4 h after infection and peaks at 12–24 h [9]. PCT is used to diagnose late-onset NS, bacterial meningitis, and other bacterial infections [10]. However, PCT detection sensitivity is low and easily affected by other factors, especially in neonates with low or very low birth weights [11]. Therefore, the ability of CRP and PCT to detect NS is not ideal.

In recent years, the proinflammatory cytokines interleukin (IL)-6 and IL-8 have been considered diagnostic markers for multiple diseases. They are highly expressed in numerous diseases, such as arthritis, psoriasis, cancer, and coronary artery disease [12,13,14]. Previous studies have reported the values of IL-6 and IL-8 in the early diagnosis of sepsis in children and newborns. Previous studies have shown that IL-6 and IL-8 are associated with sepsis in children and newborns and are closely related to sepsis severity. The level of IL-6 in umbilical cord blood is the only predictor of early-onset sepsis [15] and can be used to predict NS in the hours after birth. IL-6 has been used to diagnose neonatal clinical sepsis [16]. Moreover, IL-8 level is not associated with gestational age or birth time and has been gradually used in the early diagnosis of NS and auxiliary efficacy evaluation [17–19]. Additionally, studies have revealed that serum amyloid A (SAA) is highly expressed in adult and neonatal patients with sepsis [20–22].

There are many biological markers of NS, but the neonatal period has unique considerations, including gestational age and day, each of which has advantages and disadvantages. Therefore, the clinical application values of IL-6, IL-8, and SAA in the treatment of sepsis in premature infants need further exploration. There is no ideal single biological indicator that can be used to accurately diagnose NS early, but comprehensive indicators can complement each other. Thus, in the present study, we evaluated the diagnostic value of IL-6, IL-8, and SAA in NS patients compared with that of CRP and PCT. We used receiver operating characteristic (ROC) curves to assess the ability of IL-6, IL-8, and SAA alone and in combination with CRP and PCT to predict NS. These findings provide a valuable theoretical basis for the early diagnosis of NS.

## 2 Materials and methods

### 2.1 Study subjects

**Figure 1:**
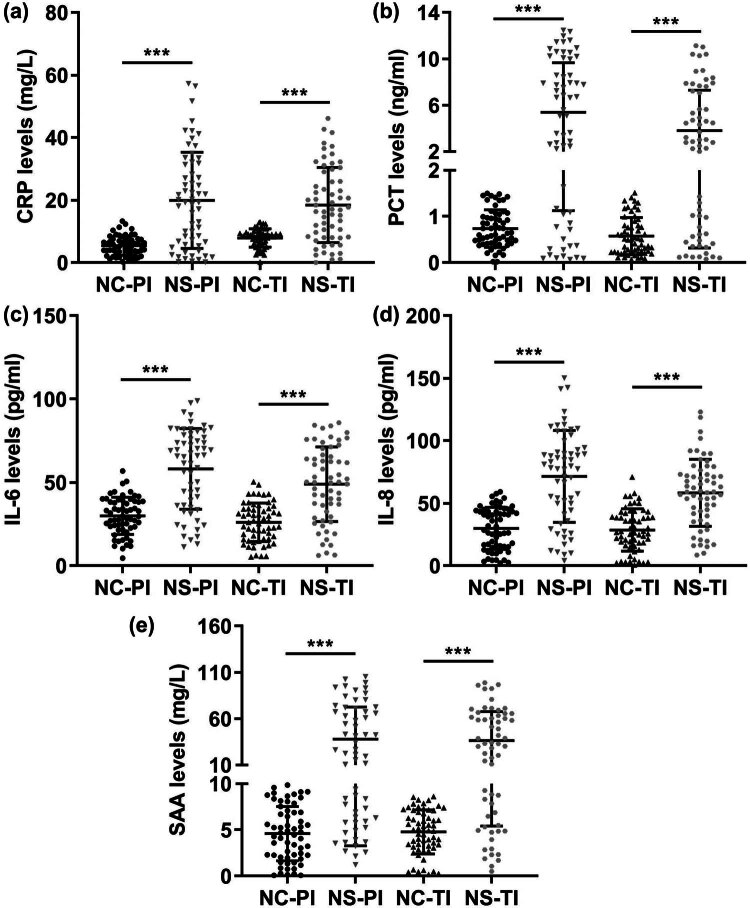
Serum CRP, PCT, IL-6, IL-8, and SAA levels in the NC-PI, NS-PI, NC-TI, and NS-TI groups. (a) Serum CRP levels were measured via latex-enhanced immunoturbidimetry. (b) Serum CRP expression was examined via the Elecsys BRAHMS PCT kit. (c) Serum IL-6. (d) IL-8 and (e) SAA expression was assessed via ELISA. ***P < 0.001.


The present study included all neonates (full-term and preterm neonates) with suspected NS treated at Children’s Hospital, Capital Institute of Paediatrics (Beijing, China) from October 2021 to June 2022. All patients were treated in accordance with the 2019 edition of the Expert Consensus on the Diagnosis and Treatment of Neonatal Sepsis, generated by the Chinese Medical Association and Professional Committee of Infectious Diseases, Neonatology Society [23]. The newborns with the following symptoms were suspected of having sepsis: abnormal body temperature (>38.5°C or <36°C), respiratory changes (average respiratory rate >2 SD above normal for age or in the presence of mechanical ventilation), lethargy, feeding difficulties, haemodynamic instability (heart rate >190 or <90 beats/min), convulsion, hypotension (blood pressure <5th percentile for this age group, or systolic blood pressure <2 SD below normal for this age group), irritability or bleeding according to the definitions for sepsis [23]. These newborns were diagnosed with sepsis upon positive blood culture. Patients were excluded from the present study if they met the following criteria: congenital deformity, toxoplasma infection during the perinatal period, prenatal viral infection, genetic disorders, immune diseases, noninformed consent, incomplete clinical data, and out-of-hospital treatment over 3 days or admission time <24 h. In addition, age- and sex-matched nonseptic patients were enrolled in the same period. The nonseptic patients were hospitalized due to neonatal jaundice. All participants were subjected to clinical and physical examinations, and their medical histories were provided by their legal guardians to rule out sepsis or sepsis risk factors. The routine examinations included assessing body temperature, respiration, heart rate, blood pressure, and bleeding, and the data were examined to analyse whether the newborns in the control group presented symptoms of suspected sepsis. Moreover, white blood cells (WBCs), neutrophils, platelets, and pathogenic bacteria were measured to confirm that they did not have an infection. Subjects with genetic disorders, noninformed consent, and incomplete clinical data were subsequently excluded. Finally, a total of 120 children with NS (60 cases of premature infants [NS-PIs] and 60 cases of term infants [NS-Tis]) and 120 noninfected participants (neonatal controls for premature infants [NC-PIs; *n* = 60] and neonatal controls for term infants [NC-TIs; *n* = 60]) were enrolled in the present study. The process of subject selection is shown in Figure 1.

**Informed consent:** Written informed consent was provided by the legal guardian of the subjects.
**Ethical approval:** The research related to human use has been complied with all the relevant national regulations, institutional policies and in accordance with the tenets of the Helsinki Declaration, and has been approved by the Ethics Committee of Children’s Hospital, Capital Institute of Paediatrics (Approval No. SHERLL2021064; Beijing, China).

### 2.2 Definition of study variables

The characteristics related to pregnancy and newborns are listed in Table 1. The variables included maternal age (year) at the date of the neonate’s birth, diagnosis of preeclampsia and chorioamnionitis during pregnancy, method of delivery, vaginal delivery/caesarean section, contamination of the amniotic fluid with meconium, newborn sex, postnatal age at admission to the neonatal intensive care unit (NICU) (day), gestational age (week), and birth weight (kg). The clinical outcomes and management during the hospital stay are presented in Table 2. The variables included hospital stay duration (day), jaundice (serum bilirubin: full-term neonates >12 mg/dl, preterm neonates >15 mg/dl), cardiopulmonary reanimation (whether newborns received any type of cardiopulmonary resuscitation during hospitalization), venous access (presence or absence of venous access established by puncture for infusion or rescue), parenteral nutrition (whether nutritional support is given intravenously), mental alterations (anxiety, irritability and/or altered consciousness), hypotension, and hypoperfusion (capillary filling >3 s).

**Table 1:** Characteristics related to pregnancy and newborns in sepsis and control groups

Pregnancy variables	NC-PI (*n* = 60)	NC-TI (*n* = 60)	NS-PI (*n* = 60)	NS-TI (*n* = 60)
Maternal age	24.25 ± 3.57	24.11 ± 3.80	23.22 ± 4.42	23.75 ± 5.12
Preeclampsia, *n* (%)	8 (13.33)	7 (11.67)	19 (31.67)^a^	18 (30.00)^b^
Chorioamnionitis, *n* (%)	1 (1.67)	0 (0.00)	16 (26.67)^b^	11 (18.33)^b^
Vaginal delivery, *n* (%)	37 (61.67)	29 (48.33)	32 (53.33)	30 (50.00)
Meconium, *n* (%)	2 (3.33)	1 (1.67)	7 (11.67)^a^	5 (8.33)^a^

Newborn variables	NC-PI (*n* = 60)	NC-TI (*n* = 60)	NS-PI (*n* = 60)	NS-TI (*n* = 60)
Males, *n* (%)	31 (51.67)	37 (61.67)	32 (53.33)	34 (56.67)
Postnatal age (days)	14.39 ± 3.85	13.92 ± 5.04	15.55 ± 6.17	15.06 ± 4.60
Gestational age (weeks)	31.71 ± 2.40	38.12 ± 0.91	31.68 ± 2.73	38.77 ± 1.16
Birth weight (kg)	2.12 ± 0.19	3.06 ± 0.35	1.98 ± 0.34^a^	3.05 ± 0.36

^a^ *P* < 0.05 and ^b^ *P* < 0.01. NC-PI, neonatal control-premature infant; NS-PI, neonatal sepsis-premature infant; NC-TI, neonatal control-term infant; NS-TI, neonatal sepsis-term infant.

**Table 2:** Clinical outcomes and management measures during the first 30 days

	NC-PI (*n* = 60)	NS-PI (*n* = 60)	NC-TI (*n* = 60)	NS-TI (*n* = 60)
Hospital stay duration (d)	12.41 ± 5.41	18.88 ± 22.21^a^	8.31 ± 4.05	17.25 ± 18.91^b^
Jaundice, *n* (%)	16 (26.67)	4 (6.67)^b^	29 (48.33)	24 (40.00)^b^
Cardiopulmonary reanimation, *n* (%)	7 (11.67)	5 (8.33)^b^	21 (35.00)	19 (31.67)^b^
Venous access, *n* (%)	30 (50.00)	25 (41.67)^b^	51 (85.00)	49 (81.67)^b^
Parenteral nutrition, *n* (%)	12 (20.00)	4 (6.67)^b^	26 (43.33)	23 (38.33)^b^
Mental alterations, *n* (%)	7 (11.67)	2 (3.33)^b^	16 (26.67)	13 (21.67)^b^
Hypotension, *n* (%)	4 (6.67)	1 (1.67)^a^	12 (20.00)	10 (16.67)^b^
Hypoperfusion, *n* (%)	4 (6.67)	1 (1.67)^a^	12 (20.00)	10 (16.67)^b^

^a^ *P* < 0.05 and ^b^ *P* < 0.01. NC-PI, neonatal control-premature infant; NS-PI, neonatal sepsis-premature infant; NC-TI, neonatal control-term infant; NS-TI, neonatal sepsis-term infant.

### 2.3 Serum sample collection

Venous blood (2 ml) was collected from all neonates with NS and negative controls on Day 1 of hospitalization. The blood was centrifuged at 4°C at 1,000 × *g* for 20 min, and the resulting supernatant was used as the serum.

### 2.4 Sample measurements

Serum CRP levels were determined using a CRP diagnosis kit (latex‐enhanced immunoturbidimetry; Yonghe, Hunan, China) according to the manufacturer’s instructions. Briefly, 2 μL of serum was incubated with 280 μL of reagent 1 at 37°C for 5 min and then with 70 μL of reagent 2 for 10 s. The absorbance was measured at 600 nm using a Hitachi 7600 automatic analyser (Hitachi, Tokyo, Japan), and after 5 min, the absorbance was measured again at 600 nm. The levels of CRP were calculated.

Serum PCT was measured using an Elecsys BRAHMS PCT kit (Roche, Basel, Switzerland) following the manufacturer’s instructions. The serum was incubated with biotinylated PCT antibody, followed by incubation with streptavidin magnetic beads. After removing the substances that were not bound to the magnetic beads, the luminescence intensity was measured using an Elecsys2010 analyser (Roche). The PCT levels were calculated.

Serum IL-6, IL-8, and SAA were detected using the human IL-6 ELISA Kit (Elabscience, Wuhan, China), the human IL-8 ELISA Kit (Elabscience), and the human SAA ELISA Kit (Abnova, Taipei, China) according to the protocol. Serum was added to the enzyme label plate and incubated at 37°C for 90 min. A biotinylated antibody working solution (100 μL) was added and incubated at 37°C for 1 h. After washing, enzyme-conjugated working solution (100 μL) was incubated at 37°C for 30 min, and TMB substrate (90 μL) was added and incubated at 37°C for 15 min. After adding 50 μL of stop buffer, the OD values were measured at 450 nm using a microplate reader (Bio-Rad, Hercules, CA, USA). The levels of IL-6, IL-8, and SAA were determined.

### 2.5 Statistical analysis

Data analysis was performed using the SPSS 20.0 software. The number of biological replicates was the sample number per group, and three technical replicates were performed. The diagnostic value was assessed via ROC curves, and the area under the curve (AUC) was calculated. The data in the tables were analysed by the chi-square test or unpaired Student’s *t*-test. The data are shown in \[\bar{x}\pm s]\] , and *P* < 0.05 was considered a statistically significant difference. In accordance with the results of previous studies, the sample size met the requirements of this study.

## 3 Results

### 3.1 Clinical characteristics of all subjects and their mothers

The clinical information of all subjects included in this study is shown in Tables 1 and 2. As shown in Table 1, the maternal age and vaginal delivery ratio of the mothers and the sex, postnatal age, gestational age, and birth weight of the newborns were not significantly different among the groups. There was a significant difference between the NS-PI and NS-TI groups and their control groups regarding preeclampsia, chorioamnionitis, and meconium. As illustrated in Table 2, there were significant differences between the NS-PI and NS-TI groups and their control groups regarding hospital stay duration, jaundice, cardiopulmonary reanimation, venous access, parenteral nutrition, mental alterations, hypotension, and hypoperfusion.

Serum CRP, PCT, IL-6, IL-8, and SAA levels in the NC, NS-PI, and NS-TI groups were analysed. The expression of CRP, PCT, IL-6, IL-8, and SAA in the serum of the NC-PI, NS-PI, NC-TI, and NS-TI subjects was analysed. The expression levels of these genes were significantly greater in NS patients than in healthy controls (Figure 1a–e).

### 3.2 Diagnostic utility of CRP, PCT, IL-6, IL-8, and SAA

#### 3.2.1 ROC curves were used to evaluate the diagnostic value of the signature

**Figure 2:**
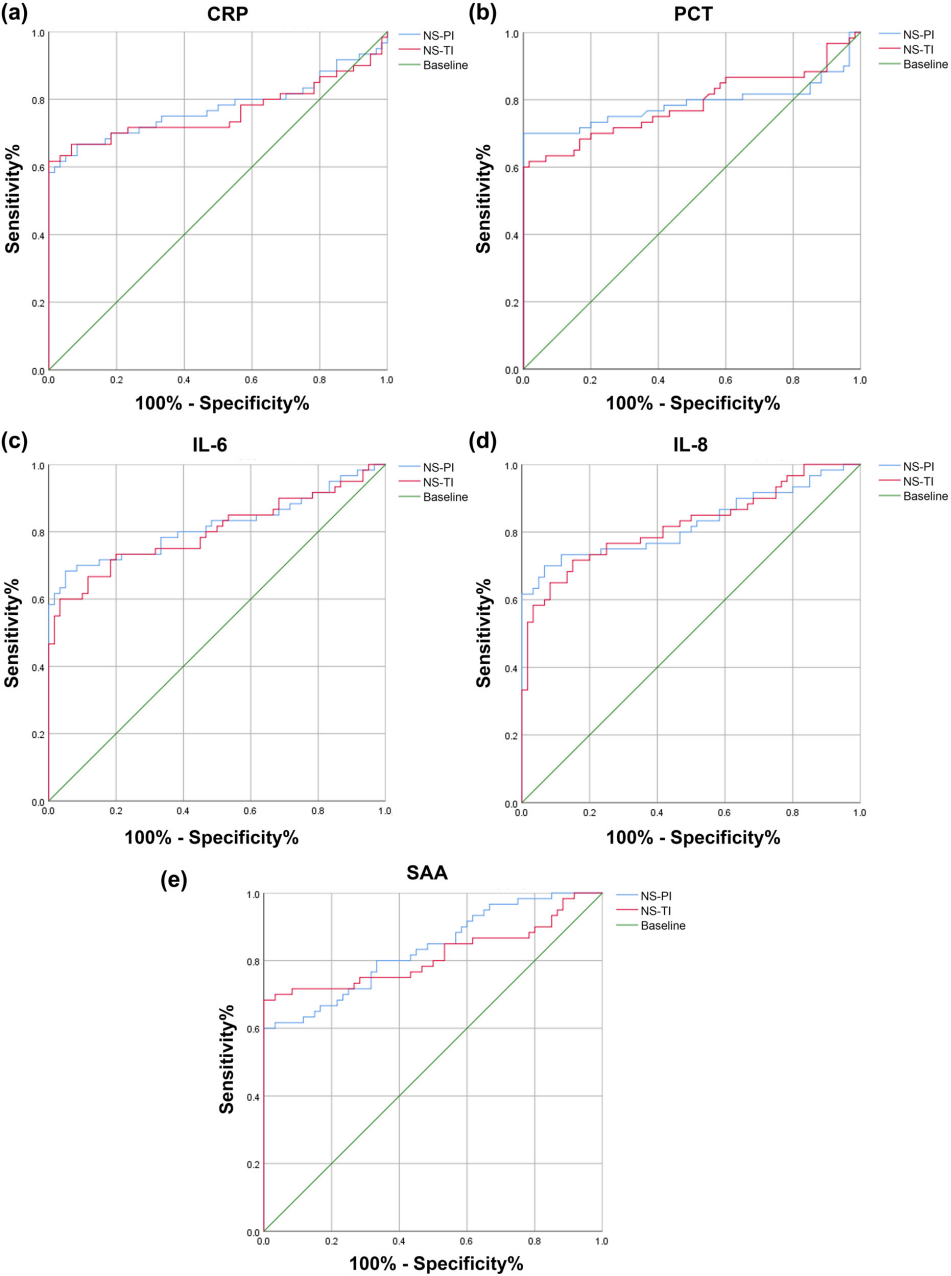
Diagnostic utility of CRP, PCT, IL-6, IL-8, and SAA. ROC curves of (a) CRP, (b) PCT, (c) IL-6, (d) IL-8, and (e) SAA levels in the NS-PI and NS-TI groups.

When the SAA cut-off value was 10.18 mg/L, the highest AUC for the diagnosis of NS-PIs was for SAA (AUC = 0.833, 95% CI 0.762–0.905; *P* < 0.001). When the CRP cut-off value was 9.562 mg/L, the smallest AUC for the diagnosis of NS-PIs was for CRP (AUC = 0.776, 95% CI 0.684–0.867; *P* < 0.001). When the IL-8 cut-off value was 52.03 pg/mL, the highest for the diagnosis of NS-TI was for IL-8 (0.821). When the IL-8 cut-off value was 52.03 pg/mL, the highest AUC for the diagnosis of NS-TI was for IL-8 (AUC = 0.821, 95% CI 0.745–0.898, *P* < 0.001). When the CRP cut-off value was 13.18 mg/L, the smallest AUC for the diagnosis of NS-TIs was for CRP (AUC = 0.762, 95% CI 0.667–0.857; *P* < 0.001) (Figure 2 and Table 3).

**Table 3:** ROC curve result analysis in Figure 2

Index	Cut-off	AUC value	Sensitivity	Specificity	PPV	NPV	Youden	ACC	95%CI	*P*
CRP (NS-PI)	9.562	0.776	0.667	0.917	0.889	0.734	0.584	0.792	0.684–0.867	＜0.001
CRP (NS-TI)	13.177	0.762	0.617	1.000	1.000	0.723	0.617	0.809	0.667–0.857	＜0.001
PCT (NS-PI)	1.567	0.788	0.7	1.000	1.000	0.769	0.700	0.850	0.694–0.881	＜0.001
PCT (NS-TI)	1.420	0.786	0.617	0.983	0.973	0.720	0.600	0.800	0.698–0.873	＜0.001
IL-6 (NS-PI)	44.487	0.817	0.683	0.950	0.932	0.750	0.633	0.817	0.736–0.898	＜0.001
IL-6 (NS-TI)	43.422	0.801	0.6	0.967	0.948	0.707	0.567	0.784	0.718–0.883	＜0.001
IL-8 (NS-PI)	52.026	0.825	0.7	0.933	0.913	0.757	0.633	0.817	0.746–0.904	＜0.001
IL-8 (NS-TI)	52.087	0.821	0.65	0.917	0.887	0.724	0.567	0.784	0.745–0.898	＜0.001
SAA (NS-PI)	10.176	0.833	0.6	1.000	1.000	0.714	0.600	0.8	0.762–0.905	＜0.001
SAA (NS-TI)	8.692	0.815	0.683	1.000	1.000	0.759	0.683	0.842	0.732–0.898	＜0.001

Note: PPV, positive predictive value; NPV, negative predictive value; ACC, accuracy.

### 3.3 Diagnostic value of the combination of CRP and IL-6, IL-8, or SAA

**Figure 3:**
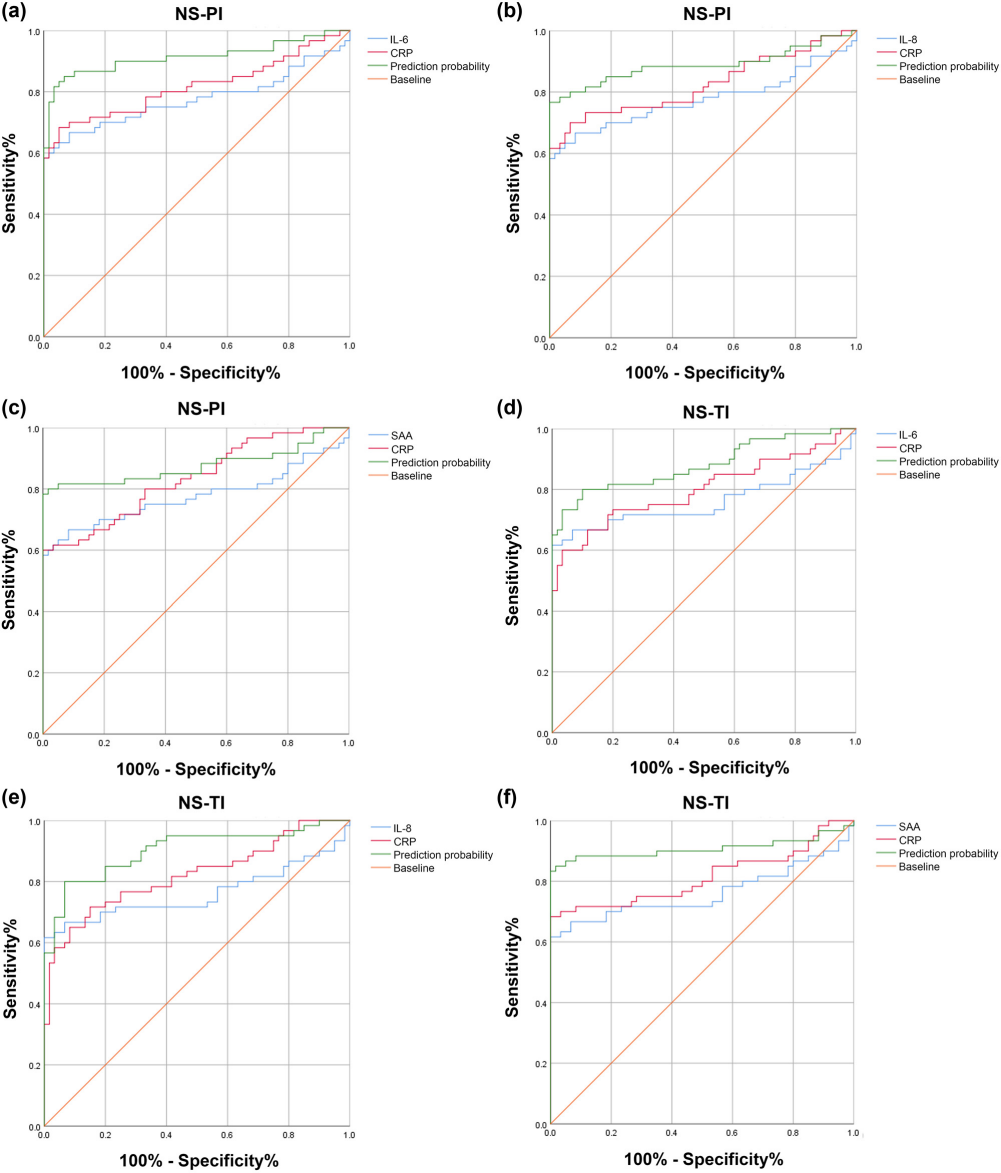
The diagnostic value of the combination of CRP and IL-6, IL-8, or SAA. ROC curves of (a) CRP combined with IL-6, (b) CRP combined with IL-8, and (c) CRP combined with SAA in the NS-PI. ROC curves of (d) CRP combined with IL-6, (e) CRP combined with IL-8, and (f) CRP combined with SAA in NS-TI patients.

The AUCs of the combinations of CRP and IL-6, IL-8, or SAA for NS-PIs were 0.913 (95% CI: 0.855–0.971), 0.887 (95% CI: 0.819–0.955), and 0.876 (95% CI: 0.805–0.948), respectively (Figure 3a–c and Table 4). The ROC curves for the combination of CRP and IL-6, IL-8, or SAA for NS-TIs exhibited AUCs of 0.881 (95% CI: 0.818–0.945), 0.902 (95% CI: 0.845–0.959), and 0.908 (95% CI: 0.942–0.974), respectively (Figure 3d–f and Table 4). The sensitivities for IL-6, IL-8, and SAA combined with CRP in the NS-PI group were 0.817, 0.767, and 0.8, respectively, whereas the sensitivities in the NS-TI group were 0.8, 0.8, and 0.85, respectively (Table 4). The specificities for IL-6, IL-8, or SAA combined with CRP were 0.967, 1.000, and 0.983 in the NS-PI group and 0.900, 0.933, and 0.983 in the NS-TI group, respectively (Table 4).

**Table 4:** ROC curve result analysis in Figure 3

Index	Cut-off	AUC value	Sensitivity	Specificity	PPV	NPV	Youden	ACC	95%CI	P值
IL-6 + CRP (NS-PI)	0.580	0.913	0.817	0.967	0.961	0.841	0.784	0.892	0.855–0.971	＜0.001
IL-8 + CRP (NS-PI)	0.595	0.887	0.767	1.000	1.000	0.811	0.767	0.884	0.819–0.955	＜0.001
SAA + CRP (NS-PI)	0.490	0.876	0.8	0.983	0.979	0.831	0.783	0.892	0.805–0.948	＜0.001
IL-6 + CRP (NS-TI)	0.450	0.881	0.8	0.900	0.889	0.818	0.700	0.850	0.818–0.945	＜0.001
IL-8 + CRP (NS-TI)	0.553	0.902	0.8	0.933	0.923	0.823	0.733	0.867	0.845–0.959	＜0.001
SAA + CRP (NS-TI)	0.370	0.908	0.85	0.983	0.980	0.868	0.833	0.917	0.842–0.974	＜0.001

Note: PPV, positive predictive value; NPV, negative predictive value; ACC, accuracy.

### 3.4 Diagnostic value of the combination of PCT and IL-6, IL-8, or SAA

**Figure 4:**
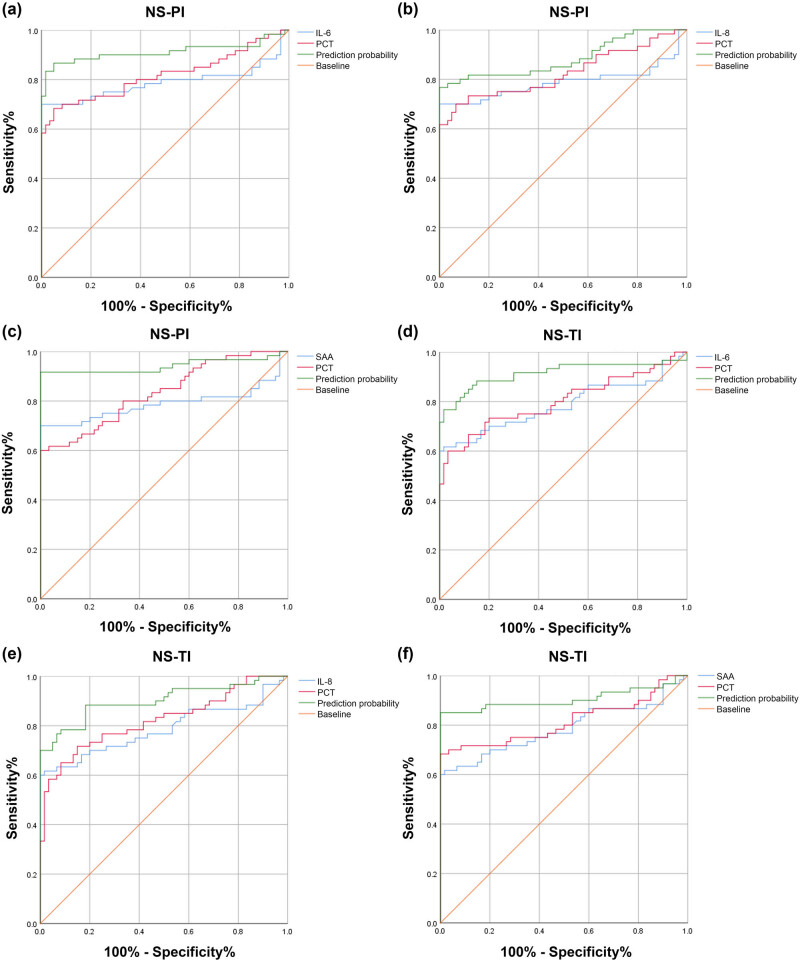
The diagnostic value of the combination of PCT and IL-6, IL-8, or SAA. Comparison of the ROC curves of (a) PCT+IL-6, (b) PCT+IL-8, and (c) PCT+SAA in the NS-PI group. Comparison of the ROC curves of (d) PCT+IL-6, (e) PCT+IL-8, and (f) PCT+SAA in the NS-TI group.

Finally, we evaluated the diagnostic value of the combination of PCT and IL-6, IL-8, or SAA. The AUCs of PCT combined with IL-6, IL-8, or SAA for NS-PIs were 0.911 (95% CI: 0.849–0.974), 0.884 (95% CI: 0.820–0.949), and 0.942 (95% CI: 0.89–0.993), respectively (Figure 4a–c and Table 5). Additionally, the AUCs of PCT combined with IL-6, IL-8, or SAA for NS-TIs were 0.914 (95% CI: 0.855–0.973), 0.901 (95% CI: 0.945–0.959), and 0.904 (95% CI: 0.84–0.969), respectively (Figure 4d–f and Table 5). The sensitivities for IL-6, IL-8, or SAA combined with CRP were 0.867, 0.767, and 0.917 in the NS-PI group and 0.767, 0.783, and 0.85 in the NS-TI group, respectively (Table 5). Additionally, the specificities for IL-6, IL-8, or SAA combined with CRP were 0.950, 0.98, and 0.98 in the NS-PI group and 0.983, 0.917, and 0.98 in the NS-TI group, respectively (Table 5).

**Table 5:** ROC curve results analysis in Figure 4

Index	Cut-off	AUC value	Sensitivity	Specificity	PPV	NPV	Youden	ACC	95%CI	*P*值
IL-6 + PCT (NS-PI)	0.446	0.911	0.867	0.950	0.945	0.877	0.817	0.909	0.849–0.974	＜0.001
IL-8 + PCT (NS-PI)	0.518	0.884	0.767	1.000	1.000	0.811	0.767	0.884	0.82–0.949	＜0.001
SAA + PCT (NS-PI)	0.523	0.942	0.917	1.000	1.000	0.923	0.917	0.959	0.89–0.993	＜0.001
IL-6 + PCT (NS-TI)	0.610	0.914	0.767	0.983	0.978	0.808	0.750	0.875	0.855–0.973	＜0.001
IL-8 + PCT (NS-TI)	0.532	0.901	0.783	0.917	0.904	0.809	0.700	0.850	0.845–0.959	＜0.001
SAA + PCT (NS-TI)	0.487	0.904	0.850	1.000	1.000	0.870	0.850	0.925	0.84–0.969	＜0.001

Note: PPV, positive predictive value; NPV, negative predictive value; ACC, accuracy.

## 4 Discussion

To date, despite the significant development of neonatal care management, the mortality rate due to NS remains high. Early screening of NS can improve patient outcomes and reduce the use of antibiotics [24]. The diagnosis of NS is slow and prone to false negatives. Although many biomarkers have been developed for the early diagnosis of NS, their clinical application effect is not ideal [25]. Haematological indices, cell adhesion molecules, and inflammatory biomarkers are more rapid diagnostic markers for NS [24]. CRP and PCT are the acute-phase reactants that are most studied for NS diagnosis. Brown et al. reported that the serum CRP level cannot be used to accurately predict late-onset NS due to pooled sensitivity [26]. Moreover, the levels of SRP are also increased in the hepatitis B vaccine response, so it is less specific for the diagnosis of NS [27]. PCT can effectively distinguish between bacterial and viral infections, and its diagnostic sensitivity for NS is greater than that of CRP [11]. However, the levels of PCT in early-onset NS may be normal, and continuous measurements are needed for a better diagnosis [28]. Therefore, novel biomarkers with increased sensitivity and specificity need to be explored for the early diagnosis of NS.

IL-6 is produced by a variety of cells, such as macrophages, T cells, and B cells. In the context of neonatal infection, hypoxia, and ischaemia, proinflammatory cytokines play a key role in the pathogenesis of tissue damage and organ failure. IL-6 is a proinflammatory factor that promotes CRP and SAA release and is a key component of the inflammatory cytokine network. IL-6 levels increase 2 h after infection, peak 4 h after infection, and return to normal 8 h after infection due to its half-life of 100 min [29]. IL-6 is a biomarker of early-onset NS and NS with premature rupture of membranes [16,30,31]. In addition, umbilical cord blood IL-6 levels have the potential to independently diagnose NS in PI patients with premature rupture of membranes [15,32]. In this study, we focused on the diagnostic utility of IL-6 in term and preterm infants and found that IL-6 levels were increased in NS. The diagnostic value of IL-6 was better than that of CRP and PCT, and the combination of IL-6 and CRP or PCT was better than that of IL-6 alone. The diagnostic sensitivity of IL-6 combined with either CRP or PCT was greater than that of IL-6 alone, whereas the specificity of IL-6 combined with CRP increased only in the NS-PI group, and the specificity of IL-6 combined with PCT increased only in the NS-TI group. These results suggest that, compared with CRP or PCT, IL-6+CRP has a high diagnostic value for NS-PIs, and IL-6+PCT has a high diagnostic value for NS-TIs.

Similar to IL-6, IL-8 is also a proinflammatory factor. IL-8 is a neutrophil chemotactic protein produced by stimulating human mononuclear macrophages. IL-8 is a multifunctional factor that specifically promotes the chemotaxis of neutrophils into inflamed tissues, promotes neutrophil degranulation, produces superoxide anions, causes respiratory outbreaks, and promotes the inflammatory response [33,34]. The content of IL-8 in noninfected newborns is very low, and when inflammation occurs, the level of IL-8 increases rapidly within 1–3 h. The half-life of IL-8 is less than 24 h, and the level of IL-8 can decrease rapidly after effective antibiotic treatment, suggesting that the level of IL-8 may be helpful for the early diagnosis of NS. The results from a meta-analysis revealed moderate accuracy of IL-8 in the diagnosis of NS [19]. In the present study, the diagnostic value of IL-8 was superior compared to that of CRP, PCT, and IL-6. Moreover, the combination of IL-8 and CRP or PCT had a better diagnostic effect than either alone for the NS-PI or the NS-TI group. The sensitivity and specificity of IL-8 combined with CRP or PCT increased, except for IL-8+PCT for the NS-TI group. IL-8+CRP has a high diagnostic value for the NS-PI and NS-TI groups but a lower diagnostic value than IL-6 combined with CRP or PCT.

After infection or injury, SAA is regulated by proinflammatory factors [34]. SAA is rapidly synthesized and released into the blood by activated macrophages and fibroblasts, replaces apolipoprotein A1 (ApoA1), and rapidly binds to high-density lipoprotein (HDL) through the N-terminus. The SAA concentration begins to increase at 3–6 h after infection, with a half-life of approximately 50 min and can reach 10–1,000 times the normal value [19]. The degree of elevation was positively correlated with the severity of the disease [22,35]. After pathogens are removed, SAA is quickly reduced to normal levels, making it a sensitive indicator of infection and inflammation recovery [36]. SAA levels in infants with suspected and confirmed sepsis are higher than those in the control group. The detection method using SAA is simple, which is more advantageous in the diagnosis of NS [37]. In addition, SAA has greater diagnostic accuracy in early-onset NS [38]. Among all the biomarkers identified in this study, serum SAA had the best diagnostic value for NS-PIs. Moreover, SAA combined with PCT had the best diagnostic effect on the NS-PI group. The sensitivity of the combination of SAA and CRP or PCT was greater than that of either alone; however, the specificity did not significantly change. In the NS-TI group, the diagnostic value of SAA+CRP was greater than that of SAA+PCT.

However, the aforementioned data are only based on a small sample of subjects and remain at the level of theoretical prediction; thus, further research is needed for clinical application, such as validation in an independent multicentre cohort.

In conclusion, the diagnostic utility of IL-6, IL-8, and SAA alone for NS is better than that of CRP and PCT alone. Moreover, PCT combined with SAA is more suitable for diagnosing NS-PIs, and PCT combined with IL-6 is more suitable for diagnosing NS-TIs. These findings provide a theoretical reference for the diagnosis of NS.
